# Impact of L-carnitine supplementation on the *in vitro* developmental competence and cryotolerance of buffalo embryos

**DOI:** 10.14202/vetworld.2021.3164-3169

**Published:** 2021-12-26

**Authors:** Mohamed M. M. El-Sokary, Al-Shimaa Al-H. H. El-Naby, Amal R. Abd El Hameed, Karima Gh. M. Mahmoud, T. H. Scholkamy

**Affiliations:** 1 Department of Theriogenology, Faculty of Veterinary Medicine, Benha University, Egypt; 2 Department of Animal Reproduction and A.I., Veterinary Research Division, National Research Centre, Dokki, Giza, Egypt; 3 Department of Field Investigations, Animal Reproduction Research Institute, Agriculture Research Center, Giza, Egypt

**Keywords:** antioxidant, buffalo embryos, cryotolerance, L-carnitine

## Abstract

**Background and Aim:**

Despite many trials, buffalo embryos have poor cryosurvivability because of their high lipid content. L-carnitine was found to be a lipid-reducing agent when added to oocyte and embryo culture media. The study aimed to determine the most effective concentration of L-carnitine to improve the oocyte developmental competence and cryotolerance of buffalo embryos.

**Materials and Methods:**

*In vitro* maturation and embryo culture media were supplemented with four concentrations of L-carnitine: 0 (control), 0.25, 0.5, and 1 mM. Good-quality embryos on 7 days were vitrified using mixtures of dimethyl sulfoxide and ethylene glycol at two concentrations (3.5 and 7 M).

**Results:**

The result showed that the cleavage and morula rates were significantly (p<0.05) higher in the 0.5 mM group. Blastocyst rates were significantly (p<0.05) higher at both 0.5 and 1 mM. The rates of viable embryos directly after thawing were significantly (p<0.05) increased in the 0.5 mM group. No significant difference was found in embryos cultured for 24 h after warming among all the groups.

**Conclusion:**

The addition of L-carnitine at a concentration of 0.5 mM to the culture media improves the oocyte developmental competence and cryotolerance of buffalo embryos directly after warming but not after 24 h of culture. Nevertheless, further studies must identify how L-carnitine exerts its beneficial micromechanisms.

## Introduction

Various reproductive biotechnologies have been adopted to improve the genetic progress of animals. The technologies established so far have been used to achieve several goals in animal production; for example, effective applications of germ cell production in females and males and international interchange of genetic material using frozen semen and embryos. Among these technologies, *in vitro* embryo production (IVEP) is an important technique that has great potential for genetic enhancement [1] through conservation of high-quality germline and transferring them over generations. During oocyte maturation and embryo culture, various factors are crucial to oocyte developmental competence, embryo quality, and cryotolerance, such as lipid content, lipid composition, embryo metabolism, apoptosis, gene expression pattern [2], optimized culture media composition (additives, salts, amino acids, hormones, sugars, antioxidants, pH, and osmolarity), temperature, oxygen tension, and oocyte donor and semen quality [3]. As an integral event during metabolism, oocytes use the energy derived from lipid droplets located in the cumulus-oocyte complex through the beta-oxidation pathway for subsequent metabolic processes [4]. In mammalian embryos, lipid metabolism, including fatty acid transportation from the cytosol to mitochondria, is an essential process for adenosine triphosphate (ATP) production during early development [4]. The unique pattern of lipid droplet distribution localizes them at the plasma membrane or close to organelles such as mitochondria and endoplasmic reticulum, which seems to be the main target for oxidative injury [5].

Among mammals, buffalo embryos have poor survivability and cryotolerance because of their high lipid content, which is considered a major limiting factor of IVEP technology [6]. During oocyte and/or embryo development or cooling, high lipid contents are believed to cause morphologic and functional damage. Such damage induces zona pellucida fractures and meiotic spindle abnormalities that cause chromosomal aneuploidy [7]. It also induces zona hardening due to premature release of cortical granules, reduced sperm penetration into oocytes and subsequent embryo development[8], damaged organelles such as mitochondria and endoplasmic reticulum [5], and reduced ATP content [9]. Eventually, the resultant changes induce the generation of reactive oxygen species (ROS), which, in turn, lead to oxidative stress that causes damage to cellular macromolecules such as nucleic acids, phospholipids, and proteins [10]. Thus, antioxidant substances should be added to the culture medium to ameliorate or prevent oxidative stress [11]. Moreover, lipid droplet removal using chemical agents improves oocyte and embryo resistance to cryopreservation [12].

Several studies have established effective ways to decrease the oocyte and embryo lipid content. Many lipid-lowering agents have been tested, including cysteamine [13], cysteine, N-acetyl-l-cysteine, catalase, superoxide dismutase [14], melatonin [15], alpha-tocopherol [16], ascorbic acid [17], and L-carnitine [18-20]. L-carnitine is a water-soluble antioxidant that plays an essential role in fatty acid metabolism in mice [21]. It also plays a key role in beta-oxidation, fatty acid transportation into mitochondria for ATP production, and mouse embryo development [11], thereby leading to increased mitochondrial activity and elevated intracellular ATP content. L-carnitine has also been shown to neutralize the embryotoxic effects of H_2_O_2_ (up to 500 mM) in mouse embryo culture medium [22]. It has been suggested that endogenous lipid metabolism plays an essential role in oocyte maturation and subsequent embryonic development in cattle [20], porcine [19], and mouse [18]. Exogenous L-carnitine is considered an enhancer of lipid metabolism in the *in vitro* development and freezing survivability of bovine embryos [23]. Moreover, L-carnitine has been shown to respond to lipid droplets distributed from the peripheral area to the inner cytoplasm in oocytes [19]. In buffalo, few studies have reported the supplementation of L-carnitine to *in vitro* maturation and embryo culture media [24-26]. However, the best dosage and the impact on embryo development and cryotolerance were controversial.

The study aimed to determine the most effective concentration of L-carnitine in the culture media, which can subsequently improve the developmental competence and cryotolerance of buffalo embryos.

## Materials and Methods

### Ethical approval

The research protocol was discussed and approved by the Council of Theriogenology Department, Faculty of Veterinary Medicine, Benha University, dated April 15, 2015.

### Study period and location

The study was carried out from October 2016 to May 2017. The research to study the impact of L-carnitine supplementation on the *in vitro* developmental competence and cryotolerance of buffalo embryos was carried out at the Department of Animal Reproduction, A.I., Veterinary Research Division at National Research Centre, Dokki, Giza, Egypt and the Department of Field Investigations, Animal Reproduction Research Institute, Agriculture Research Center, Giza, Egypt.

### Oocyte collection and selection

The methods used for oocyte recovery and selection in buffaloes have been described by Mahmoud *et al*. [27]. Briefly, ovaries were collected from apparently healthy females directly after slaughter. The ovaries were transported from the local slaughterhouse to the laboratory in a modified phosphate buffer saline at pH 7.2 containing penicillin (100 IU/mL) and streptomycin (100 μg/mL). Cumulus oocytes that were aspirated from follicles 2-8 mm in diameter using a 10 mL syringe with an 18-gauge needle were washed in TCM-199, serum (10%), and gentamicin (50 μg/mL). Good-quality oocytes with multilayers of cumulus cells and a homogenous cytoplasm were used in the experiments.

### *In vitro* maturation

Oocyte maturation was conducted according to the method reported by Mahmoud *et al*. [28]. The selected oocytes were supplemented with four concentrations of L-carnitine: 0 (control), 0.25, 0.5, and 1 mM. The oocytes were then cultured in 100 mL droplets of maturation medium containing TCM-199, 10% calf serum, and 50 mg/mL gentamycin. The oocytes were then covered with protective mineral oil and incubated for 24 h at 38.5°C with 5% CO_2_.

### *In vitro* fertilization and culture

We treated spermatozoa as described by Niwa and Ohgoda [29]. Approximately two semen straws of frozen buffalo were thawed for 1 min in a water bath at 35°C-37.8°C. The spermatozoa were centrifuged (800 × *g* for 10 min) twice in a BO medium [30] containing 10 mg/mL heparin and 2.5 mM of caffeine without bovine serum albumin (BSA). The pellets were diluted with a BO medium containing BSA (20 mg/mL) to adjust the concentration of spermatozoa to 12.5 × 10^6^ sperm/mL. Mature oocytes were washed multiple times in a BO medium containing 10 mg/mL BSA. At that point, the oocytes were brought into 100 mL droplets of sperm suspension under paraffin oil; the spermatozoa and oocytes were cocultured for 5 h under culture conditions (5% CO_2_, 38.5°C, 95% humidity). Then, the oocytes were washed in TCM-199 to eliminate attached spermatozoa. The oocytes were again replaced with the previously prepared coculture 100 mL droplets consisting of TCM-199 + 10% serum and the four concentrations of L-carnitine (0, 0.25, 0.5, and 1 mM). Cleavage was recorded after 3 days of culture, and the numbers of morula on day 5 and blastocyst stages on day 7 were evaluated.

### Embryo vitrification and warming

A vitrification solution (VS) was prepared in TCM 199 containing 2.5 mM of HEPES + 20% fetal calf serum (FCS). The embryos were vitrified in 0.25 mL straws following 2-step addition of a cryoprotectant. The embryos were placed in VS1: 1.75 M ethylene glycol (EG) + 1.75 M dimethyl sulfoxide (DMSO) for 2.5 min. The embryos were then placed in 7 M of VS2: 3.5 M EG + 3.5 M DMSO for 45 s. The mini-straws were placed in liquid nitrogen vapor for 1 min and then plunged into a liquid nitrogen tank for 1 month. At warming, straws were held for 10 s in the air, placed in water for 30 s at 37°C, and flicked several times to mix columns. The embryos were washed in 0.5 M galactose at 20°C-22°C for 5-10 min, washed 5 times in TCM with 5% FCS, and cultured at 38.5°C and 5% CO_2_ for a further 24 h.

### Survival assay

The embryos were evaluated morphologically for viability directly after warming and cultured *in vitro* for 24 h. The embryos that developed to more advanced stages, with obviously noticeable inner cell mass, were considered to have survived. Morulae that developed into blastocysts and the blastocysts that expanded were defined as surviving.

### Experimental design

#### Experiment 1

The first experiment was carried out to study the effect of different concentrations of L-carnitine on the developmental competence of buffalo embryos. Immature oocytes were classified into four groups, each supplemented with 0 (control), 0.25, 0.5, and 1 mM of L-carnitine. Mature oocytes were fertilized, and presumptive zygotes were cultured in the four concentrations of L-carnitine. The developmental competence of embryos was recorded at different points based on the calculation of cleavage, morula, and blastocyst formation rates.

#### Experiment 2

The second experiment was carried out to study the effect of different concentrations of L-carnitine on the cryotolerance of buffalo embryos. All good-quality embryos on 7^th^ day were vitrified using mixtures of DMSO and EG in TCM-199 with two concentrations of cryoprotectants (3.5 and 7 M). The four groups (0, 0.25, 0.5, and 1 mM) of vitrified embryos were thawed, and the survival rate was assessed directly after thawing and after a further 24 h.

### Statistical analysis

Three replicates per group were statistically analyzed with analysis of variance using. IBM SPSS Statistics for Windows, Version 16.0. (IBM Corp., NY, USA) Comparison among the means was performed by LSD and Duncan’s multiple range test. Differences were significant at p<0.05 level.

## Results

### Experiment 1

The effect of different concentrations of L-carnitine on the developmental competence of buffalo oocytes after culture was studied and we found that, embryo development represented by cleavage, morula, and blastocyst rates was improved by the addition of L-carnitine compared with control. The cleavage and morula rates were significantly (p<0.05) higher in the group with 0.5 mM L-carnitine compared with that with other concentrations (Table-1). Meanwhile, there was no significant difference between 0.25 and 1 mM of L-carnitine concentrations. In addition, blastocyst rates were significantly (p<0.05) higher in the 0.5 and 1 mM groups than in the other groups.

**Table 1:** Developmental competence of buffalo oocytes after culture supplementation with different concentrations of L-carnitine (Mean±SE).

L-carnitine concentration	Total no. of inseminated oocytes	Cleavage	Morula	Blastocyst
		
		No. (%)	No. (%)	No. (%)
0 mM	91	50 (54.8±1.6)^b^	34 (36.9±2.6)^b^	13 (14.6±1.1)^b^
0.25 mM	81	48 (59.6±2.0)^ab^	31 (39.1±5.1)^ab^	16 (19.7±0.9)^ab^
0.5 mM	76	52 (66.7±4.2)^a^	37 (48.3±0.8)^a^	17 (21.7±1.8)^ab^
1 mM	56	36 (63.5±3.2)^ab^	25 (43.9±3.3)^ab^	13 (24.3±3.8)^a^

Percent of total inseminated oocytes. No. = number.

a ,

b Values inside similar column without common superscripts differ p*<*0.05, three replicates (ANOVA test). ANOVA=Analysis of variance, SE=Standard error

### Experiment 2

The effect of different concentrations of L-carnitine on cryotolerance of buffalo embryos was studied and we found that the rates of viable embryos directly after thawing were significantly (p<0.05) higher in the 0.5 mM group than in the other L-carnitine concentration groups (Table-2). However, the percentages of viable embryos cultured for 24 h after warming did not differ significantly among all the groups.

**Table 2:** Cryotolerance of buffalo embryos after culture supplementation with different concentrations of L-carnitine (Mean±SE).

L-carnitine concentration	Total no. of inseminated oocytes	Cleavage	Morula	Blastocyst
		
		No. (%)	No. (%)	No. (%)
0 mM	91	50 (54.8±1.6)^b^	34 (36.9±2.6)^b^	13 (14.6±1.1)^b^
0.25 mM	81	48 (59.6±2.0)^ab^	31 (39.1±5.1)^ab^	16 (19.7±0.9)^ab^
0.5 mM	76	52 (66.7±4.2)^a^	37 (48.3±0.8)^a^	17 (21.7±1.8)^ab^
1 mM	56	36 (63.5±3.2)^ab^	25 (43.9±3.3)^ab^	13 (24.3±3.8)^a^

a ,

b Values inside similar column without common superscripts differ p*<*0.05, three replicates (ANOVA test). ANOVA=Analysis of variance, SE=Standard error

## Discussion

Several factors have been proposed to affect oocyte developmental competence and embryo cryotolerance [31]. One crucial factor is the large lipid droplets inside the cytoplasm of the oocytes that assist in energy production. These droplets were found to level up the sensitivity of oocytes and embryos during vitrification [32]. Throughout oocyte and/or embryo development or cryopreservation, high lipid cargo is supposed to lead to morphologic, structural, and functional damage. Specifically, fractures of the zona pellucida and meiotic spindle abnormalities cause chromosomal aneuploidy [7]. Moreover, changes in lipid droplets trigger zona hardening and premature diffusion of cortical granules [8], damage organelles such as mitochondria and endoplasmic reticulum [5], and reduce ATP content [9]. Eventually, the resultant changes induce the generation of ROS, which, in turn, lead to oxidative stress that causes damage to cellular macromolecules such as nucleic acids, phospholipids, and proteins [10]. In embryos, high intracellular ROS levels cause cellular damage, including DNA fragmentation and apoptosis, leading to a developmental block and embryo fragmentation [33]. An imbalance between the formation of ROS and antioxidant capacity [34] can lead to many pathological effects, such as lipid peroxidation, ATP depletion, mitochondrial alterations, and fetal growth arrest. Increased ROS levels due to enhanced lipid metabolism could be ameliorated by free radical scavenging agents such as L-carnitine [35].

L-carnitine is a water-soluble antioxidant that plays a crucial role in the metabolism of fatty acids and decreases the frequency of apoptosis in animal cells [21]. In addition, L-carnitine is well known for its role in beta-oxidation, ATP production, and decreasing the lipid content during embryo development, with end results of improved cryo-survivability [11].

In this study, the cleavage and morula rates were significantly (p<0.05) higher in the group with 0.5 mM of L-carnitine. In addition, the blastocyst rates were significantly (p<0.05) higher in both 0.5 and 1 mM groups than in the other groups. Our findings were in accordance with those of Boccia *et al*. [24] and Verma *et al*. [25], who showed that the addition of L-carnitine to the culture media enhances the developmental competence of buffalo oocytes, likely by reducing the ROS level in embryos. Moreover, during *in vitro* maturation, L-carnitine was found to enhance mitochondrial activity in porcine oocytes [19] and bovine embryos [36]. L-carnitine also enhanced the level of ATP in 2-cell embryos but not at the 8-cell or blastocyst stage in bovine [37] and mice blastocysts [18].

Concerning the concentration of L-carnitine in the current work, the blastocyst rates were significantly (p<0.05) higher in both the 0.5 and 1 mM groups than in the other groups. Recently, Liang *et al*. [26] reported that buffalo oocytes treated with 0.6 mg/mL of L-carnitine during *in vitro* maturation had significantly higher rates of blastocyst formation than controls. Similar to our results, Khanmohammadi *et al*. [38] found that the developmental rate of 1-cell embryos to the blastocyst stage and the expansion of mouse blastocysts were observed in a medium supplemented with 0.5 mM of acetyl L-carnitine.

We next sought to establish the effect of different micromolar concentrations of L-carnitine at 0.25, 0.5, and 1 mM on the cryosurvivability of embryos. The obtained results suggested that the addition of 0.5 mM of L-carnitine to the culture media is of a significant benefit to the developmental competence of buffalo embryos, as evidenced by the high rate of cleavage as well as morula and blastocyst formation. The current study results indicate that the rates of viable embryos directly after thawing were significantly (p<0.05) higher in the 0.5 mM group than in the other groups. The improvement of cryotolerance of the embryos directly after thawing may be through enhancing lipid metabolism. Moreover, during the *in vitro* culture of buffalo embryos, it has been suggested that 0.25 mM of L-carnitine supplementation enhances blastocyst development and cryotolerance resistance [24]. However, the percentages of viable embryos that were cultured for 24 h after warming did not differ significantly among all L-carnitine concentrations. Interestingly, Chankitisakul *et al*. [37] reported that supplementation of IVM media with 0.6 mg of L-carnitine had no significant effect on the embryo developmental competence of IVM oocytes after vitrification.

Taken together, the enhanced maturation of oocyte and embryonic development by the addition of L-carnitine may be attributed to two main theories. First, the metabolism of lipid through the ­beta-oxidation pathway produces ATP, which, in turn, is responsible for cytoplasmic maturation and meiotic resumption [35]. Second, L-carnitine acts as an antioxidant (ROS scavenger) by blocking oocyte and embryo degenerative changes [39]. However, the exact mechanism of the action of L-carnitine requires further study to illustrate the underlying events that lead to improved maturation and embryonic developmental competence of buffalo species.

## Conclusion

The supplementation of 0.5 mM of L-carnitine to the culture media improved buffalo embryo developmental competence and cryosurvivability directly after thawing. Moreover, L-carnitine supplementation at 0.25, 0.5, and 1 mM did not enhance the development of vitrified embryos after 24 h of culture.

## Authors’ Contributions

MMME, AAHE, ARAE, THS, and KGMM: Collected the samples and performed laboratory tests. KGMM: Designed the experiment and analyzed the data. MMME, KGM, and ARAE: Drafted and revised the manuscript. All authors read and approved the final manuscript.
